# Risk for Femoral Fractures in Parkinson’s Disease Patients with and without Severe Functional Impairment

**DOI:** 10.1371/journal.pone.0097073

**Published:** 2014-05-22

**Authors:** Petra Benzinger, Kilian Rapp, Walter Maetzler, Hans-Helmut König, Andrea Jaensch, Jochen Klenk, Gisela Büchele

**Affiliations:** 1 Department of Clinical Gerontology and Rehabilitation, Robert Bosch Krankenhaus Stuttgart, Stuttgart, Germany; 2 Institute of Epidemiology and Medical Biometry, Ulm University, Ulm, Germany; 3 Hertie-Institute for Clinical Brain Research, University Hospital Tuebingen, Tuebingen, Germany; 4 DZNE, German Center for Neurodegenerative Diseases, Tuebingen, Germany; 5 Department of Health Economics and Health Services Research, Hamburg Center for Health Economics, University Medical Center Hamburg-Eppendorf, Hamburg, Germany; Oslo University Hospital, Norway

## Abstract

**Background:**

Impaired balance is a major problem in patients with idiopathic Parkinson’s disease (PD) resulting in an increased risk of falls and fall-related fractures. Most studies which analyzed the risk of femoral fractures in patients with idiopathic PD were performed either in specialized centers or excluded very frail patients. The current study used a large population-based dataset in order to analyze the risk of femoral fractures in patients with idiopathic PD.

**Methods:**

Data from more than 880.000 individuals aged 65 years or older and insured between 2004 and 2009 at a large German health insurance company were used for the analyses. Persons with idiopathic PD were identified by the dispensing of Parkinson-specific medication and by hospital diagnoses, if available. People without PD served as the reference group. Incident femoral fractures were obtained from hospital diagnoses. Analyses were stratified by gender and information on severe functional impairment (care need) as provided by reimbursement claims.

**Results:**

Compared with the reference group, persons with idiopathic PD had a more than doubled risk to sustain a femoral fracture. The risk was higher in men (HR = 2.61; 95%-CI: 2.28–2.98) than in women (HR = 1.79; 95%-CI: 1.66–1.94). The increased risk was only observed in people without severe functional impairment. The sensitivity analysis using a refined definition of idiopathic PD patients yielded similar results.

**Conclusion:**

The findings confirm the increased risk of femoral fractures in patients with idiopathic PD. The relative risk is particularly high in male PD patients and in patients without severe functional impairment.

## Introduction

Parkinson’s disease (PD) is the second most common neurodegenerative disorder after Alzheimer’s disease [1]. Postural instability and impaired balance is one of the cardinal symptoms of PD posing these patients at an increased risk of falling [2]–[7]. Fall-related injuries represent the most frequent reason for hospital admission in PD [8]. The occurrence of falls in patients with PD has been shown to be associated with increasing age, severity of symptoms, and use of dopaminergic drugs [3], [4], [7], [9].

The increased risk of falling in combination with a low bone mineral density is putting PD patients at high risk for osteoporotic fractures [10]. In several studies, PD patients were found to have a high rate of fractures [2], [11]–[16]. Among these, femoral fractures are the most common type of non-vertebral fractures [7], [12]–[14], [16]. Femoral fractures are associated with high morbidity, mortality, and costs in the general older population [17]–[19]. In PD patients, femoral fractures are associated with a particularly high risk of unfavorable outcomes such as admission to nursing homes [20]. However, estimates of the magnitude of these patients’ increased risk are mainly based on observational studies with a limited number of PD patients and only a low number of femoral fractures [13]–[16].

PD is characterized by loss of functional abilities as the disease progresses [21]–[23]. However, the influence of functional limitations on the excess risk for femoral fractures in PD patients has not been explored so far. A large German population-based dataset containing routine data allowed us to identify older individuals with severe functional limitations in activities of daily living. The aim of this study was (1) to estimate the risk of idiopathic PD patients to sustain a femoral fracture and (2) to stratify this estimate for the presence of severe functional impairment.

## Materials and Methods

### Data Source

The routine data collection systems of the largest health insurance company in Bavaria, the *Allgemeine Ortskrankenkasse Bayern* (AOK Bavaria), was used to select data on gender, age, long term care need, admission to hospital, admission and discharge diagnosis as well as dispensed medication. Health insurance and long term care insurance is statutory in Germany. The AOK Bavaria covers nearly 50% of the population aged 65 years and over in Bavaria, a federal state with 12.5 million inhabitants.

### Data on Medication to Treat PD

Within the stored data of the AOK Bavaria, all prescriptions for medication for the treatment of PD were identified (Tab. 1). Substances were categorized according the Anatomical Therapeutic Chemical Classification System (ATC code) to substance classes. In Germany, outside hospitals Parkinson medication is available only at pharmacies with a written prescription by a physician and is reimbursed by the person’s health insurance. For reimbursement, a person’s insurance number along with type, dose and amount of drug prescribed, as well as prescription date is transferred to the health insurance. No information on the dosage regime or diagnosis is transferred. Data on reimbursement held by health insurances give complete information on all prescriptions filled in.

**Table 1:** Medication considered as treatment for PD.

	ATC codes
**Levodopa**	N04BA01/N04BA02
**Dopamine agonists**	
Apomorphine	N04BC07
Bromocriptine	N04BC01
Cabergoline	N04BC06
Lisurid	N04BC10
Pergolide	N04BC02
Pramipexole	N04BC05
Ropinirole	N04BC04
Rotigotine	N04BC09
**Anticholinergics**	
Biperiden	N04AA02
Bornaprine	N04AA11
Methixine	N04AA03
Procyclidine	N04AA04
Trihexphenidyl	N04AA01
**Budipine**	N04BX03
**COMT inhibitor**	
Entacapone	N04BX02
Tolcapone	N04BX01
**MAO B inhibitors**	
Rasagiline	N04BD02
Selegiline	N04BD01
**Amantadine**	N04BB01
**Stalevo**	N04BA03

### Study Population

**Figure 1:**
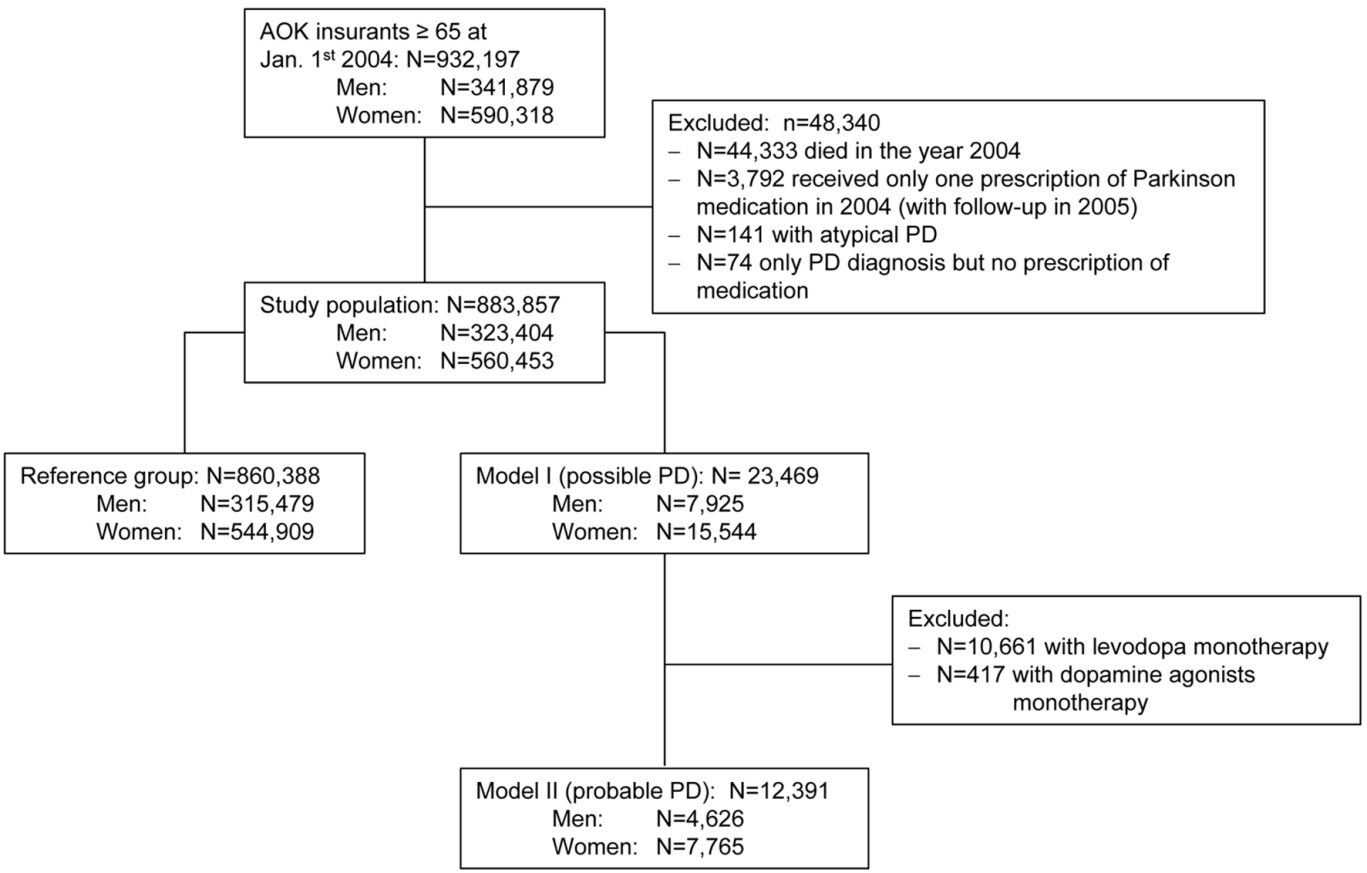
Flow chart of study population and model definitions. Identification was based on characteristics in 2004 as provided by the health insurance company (AOK: Allgemeine Ortskrankenkasse Bavaria) PD: Parkinson’s disease; N: number of participants.

The dataset consisted of 932,197 people aged 65 years and over who were member at AOK Bavaria between January 1^st^ 2004 and June 30^th^ 2009. Data from the year 2004 (January 1^st^ to December 31^st^) regarding admission or discharge diagnoses as well as PD medications were used to identify patients with idiopathic PD. Individuals dying in 2004 were not considered (N = 44,333). Identification of the study population and sub-groups is displayed in figure 1.

### Identification of Individuals with Idiopathic PD

Identification of patients with PD using routine data is not straightforward. In our dataset we could use two types of information to identify PD patients: (1) primary hospital admission and discharge diagnoses of hospitalized patients, and (2) information about antiparkinson medication dispensed by pharmacies outside the hospital. Hospital admission and discharge diagnoses covered only a small percentage of PD patients. Therefore, the main information to identify patients with idiopathic PD was the medication to treat PD.

We defined individuals as patients with an idiopathic PD if a) antiparkinson medication was dispensed at least twice in 2004, or once in 2004 and at least once in 2005 or b) idiopathic PD was the primary hospital admission or discharge diagnosis in 2004 (ICD-10: G20) and antiparkinson medication was dispensed at least once in 2004.

Most patients with idiopathic PD are treated with antiparkinson drugs and are covered by the chosen method. However, some other disease entities are also treated by antiparkinson drugs like atypical Parkinsonism, secondary Parkinsonism or restless legs syndrome (RLS). Therefore, these individuals were excluded if they could be identified by hospital admission or discharge diagnoses (ICD-10) (G21/G22/G23/G25.81). The final dataset comprised 323, 404 men and 560, 453 women.

This dataset still included patients with atypical Parkinsonism, secondary Parkinsonism or RLS. Atypical Parkinsonism and secondary Parkinsonism are very rare diseases. RLS, however, has a higher prevalence and might account for a relevant percentage of the dispensed antiparkinson medication. Medically treated RLS is usually treated by monotherapy with dopaminergic drugs. Therefore, we applied two different models to perform a sensitivity analysis. Model I (‘possible’ idiopathic PD patients) used data of all patients with antiparkinson medication (see above). Most patients with a RLS are still included in this model. Model II (‘probable’ idiopathic PD patients) excluded all individuals (n = 11,078) from model I who received levodopa only or dopamine agonists only (change of substance within and between substance class possible). In this model most patients with a RLS but also patients with an idiopathic PD treated by monotherapy with dopaminerigic drugs are excluded. Therefore, model II may over-represent patients with an advanced idiopathic PD.

Please note that these terms are not synonymous with the terms for clinical definition of PD [24] and are therefore put in brackets throughout this article.

Reference group: The reference group comprised of individuals without intake of antiparkinson medication and no hospital admission or discharge diagnosis of atypical Parkinsonism, secondary Parkinsonism or RLS in 2004.

### Definition of Severe Functional Impairment

The need for long-term nursing care was used as a marker for severe functional impairment and was assessed at the beginning of the observation period (January 1^st^ 2005). In Germany, most persons with a minimum of six months of need for nursing care are eligible to receive reimbursement for long-term care by the long-term care insurance. The long-term care insurance was introduced in the German social insurance system in 1995. All employed citizens are members by law [25]. Experts confirm a person’s eligibility to receive long-term care benefit. Long-term care benefits are granted for professional, family, and/or informal help. In order to claim long-term care benefits, people must have a daily minimum of 90 minutes of assistance with basic activities of daily living (ADL) such as washing, eating, or dressing, and instrumental activities of daily living (IADL) such as cleaning or shopping. This information thus defines relatively well functional impairment, independent of underlying diseases.

### Identification of Femoral Fractures

Hospital discharge diagnoses were used to identify femoral fractures (ICD-10: S72). The observation period started on January 1^st^ 2005 and ended on June 30^th^ 2009. Hospital admissions coding a femoral fracture that occurred within less than 30 days to a previous fracture were excluded in order to avoid double coding.

### Statistics

The accumulation of the individual person-years of individuals with PD and without (reference group) started at beginning of observation period (January 1^st^ 2005) and ended with censoring due to death or at the end of the observation period. Fracture rates and 95% confidence intervals were estimated per 1000 person-years and adjusted for age using negative binomial regression models. To quantify the relative risk for the first femoral fracture during observation time in people with ‘possible’ or ‘probable’ idiopathic PD compared to people without PD hazard ratios with 95% confidence intervals were calculated. In the applied proportional hazards models age of participants was used as ‘survival time’-variable to adjust for age and to account for the fact that the participants were of different ages at study begin. All models were stratified for sex and considered severe functional impairment by stratification. All statistical calculations were carried out using SAS version 9.3 (SAS Institute Inc., Cary, NC, USA).

### Ethics

The study was approved by the Ethical committee of Ulm University.

## Results

A total of 883,857 individuals aged 65 years and older were included in our study population. The median follow-up was 4.5 years. As ‘possible’ PD patients (model I) 23,469 individuals (2.7%) were identified and 12,391 (1.4%) as ‘probable’ idiopathic PD patients (model II). Table 2 shows baseline characteristics of the reference group as well as of individuals with ‘possible’ PD (model I) and ‘probable’ PD (model II). In both models, PD-patients were older than the reference group (77.9, 76.9 years vs. 74.4 years in individuals with ‘possible’ PD, ‘probable’ PD, and in individuals of the reference group, respectively). A higher proportion of these individuals had severe functional impairment (47.7, 50.3 and 12.7% of the population with ‘possible’ PD, ‘probable’ PD, and of individuals of the reference group, respectively). However, the median age of individuals with severe functional impairment was younger in both models compared to the reference group (81.2/79.6 years vs. 83.5 years, as for individuals with ‘possible’ PD, ‘probable’ PD, and for individuals of the reference group, respectively).

**Table 2:** Characteristics of the study population.

	Reference group	PD medication	
		Model I (possible PD)	Model II (probable PD)
Total [N (%)]	860,388 (97.3%)	23,469 (2.7%)	12,391 (1.4%)
Age (y) [Median, IQR]	74.4 (69.7–80.2)	77.9 (72.7–83.1)	76.9 (72.0–82.0)
Women (%)	63.3	66.2	62.7
Follow-up (y) [Median, IQR]	4.50 (4.50–4.50)	4.50 (2.40–4.50)	4.50 (2.47–4.50)
Number of femoral fractures (N)	33,228	1,633	919
Persons with 1 femoral fracture (N)	28,939	1,407	785
Persons with ≥2 femoral fractures (N)	2,104	110	66
Persons without care need [N (%)]	750,806 (87.3%)	12,263 (52.3%)	6,153 (49.7%)
Age (y) [Median, IQR]	73.4 (69.3–78.6)	75.1 (70.7–79.9)	74.5 (70.2–79.0)
Women (%)	61.6	64.9	60.3
Persons with care need [N (%)]	109,582 (12.7%)	11,206 (47.7%)	6,238 (50.3%)
Age (y) [Median, IQR]	83.5 (77.9–89.4)	81.2 (76.0–85.3)	79.6 (74.6–84.0)
Women (%)	75.4	67.7	65.0

PD: Parkinson’s disease; N: Number; y: Years, IQR: Inter quartile range.

Characteristics of study population at start of the observation period (January 1^st^ 2005) and number of fractures between January 1^st^ 2005 and June 30^th^ 2009.

As displayed in Table 3, the most commonly prescribed medication of individuals with ‘possible’ PD was levodopa either alone or in combination (79.0%). Only 1.8% of individuals received dopamine agonists alone and 9.4% in combination.

**Table 3:** Frequency of prescribed Parkinson medication of persons identified with ‘possible’ Parkinson’s diseases (PD).

	Number (%)*
Monotherapy	
Levodopa†	10,880 (46.4%)
Dopamine agonists†	425 (1.8%)
Others§	4,249 (18.1%)
Combinations	
Levodopa + dopamine agonists	2,074 (8.8%)
Levodopa + others§	2,951 (12.6%)
Dopamine agonists + others§	136 (0.6%)
Other combinations	2,754 (11.7%)

*percentage refers to all persons included in model I (‘possible’ PD).

† model II (‘probable’ PD) excludes persons treated with levodopa only or dopamine agonists only.

§ Others: amantadine, MAO B inhibitors, anticholinergic agents, budipine.

**Figure 2:**
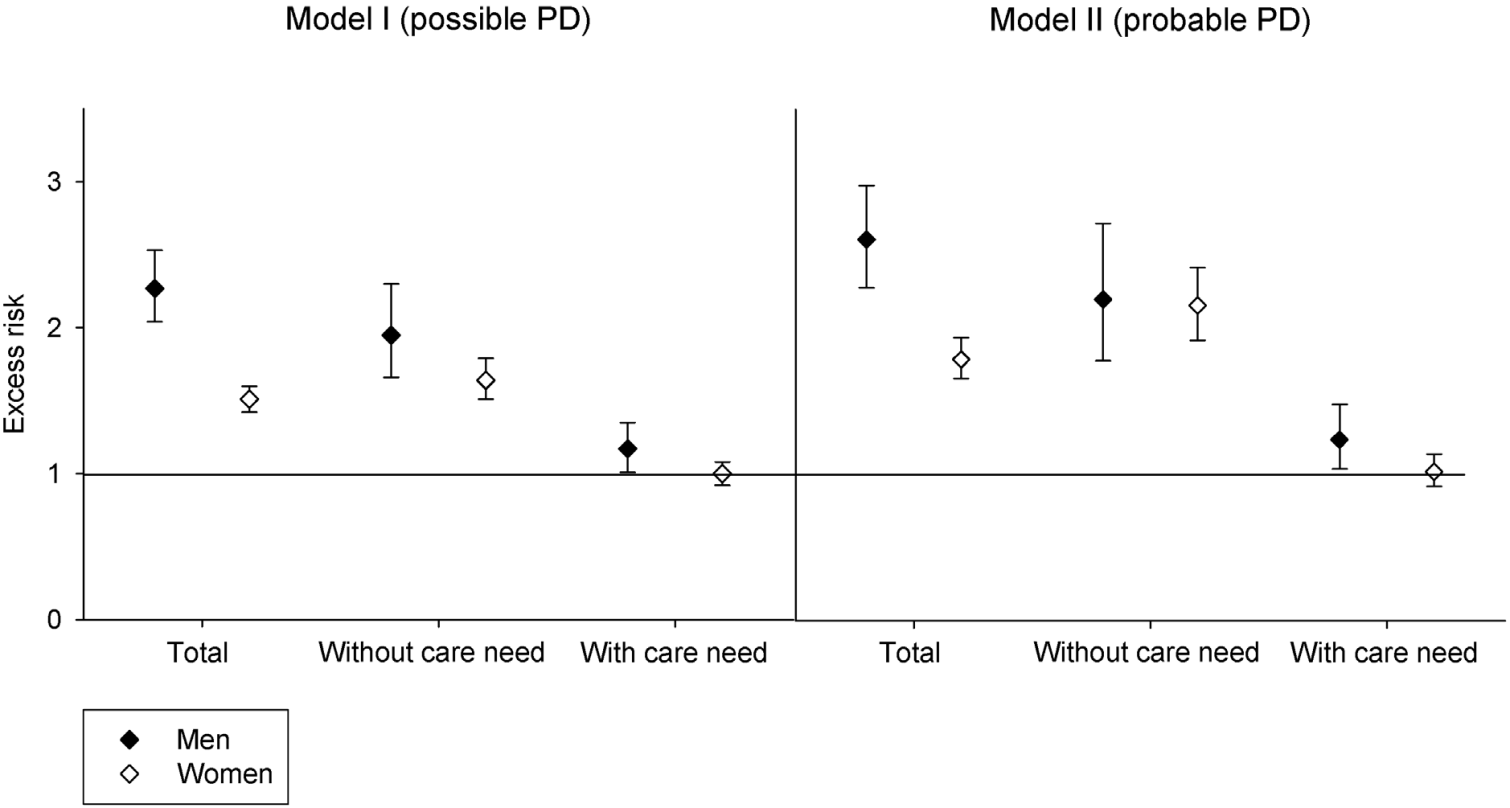
Age-adjusted risk of a first femoral fracture during a median observation period of 4.5 years. Individuals with ‘possible’ (N = 23,469) or ‘probable’ (N = 12,391) idiopathic Parkinson’s disease (PD, for definition see text) were compared to 860,388 people without PD, stratified by gender and care need.

Estimates of fracture rates were based on 34,861 femoral fractures overall. Table 4 shows age adjusted fracture rates stratified by severe functional impairment and gender. Rates for femoral fractures are ranging from 3/1000 to 33/1000 person-years. In all strata and models, women had a higher fracture rate than men. The relative difference was less pronounced in individuals with ‘possible’ or ‘probable’ idiopathic PD with severe functional impairment. Figure 2 illustrates hazard ratios (HR) using the same strata. Narrowing the criteria from model I to model II increased the HR from 2.27 to 2.61 in men, and from 1.51 to 1.79 in women. The HR was higher in men than in women in both models (HR 2.27 vs. 1.51/2.61 vs. 1.79 in model I/II, respectively). In individuals without severe functional impairment, the relative fracture risk was higher than in those with functional impairment. In fact, women with ‘probable’ idiopathic PD and severe functional impairment did not have an increased risk to sustain a femoral fracture compared to women of the reference group. Men with ‘probable’ idiopathic PD and functional impairment had a low risk increase for femoral fractures of 24%. Both models showed similar results (Fig. 2).

**Table 4:** Age-adjusted femoral fracture rates (per 1,000 person-years) of persons aged 65 and older.

	Total			Without care need			With care need		
	No. of femoral fractures	Person-years	Femoral fractures/1000 person-years (95% CI^†^)	No. of femoral fractures	Person-years	Femoral fractures/1000 person-years (95% CI^†^)	No. of femoral fractures	Person-years	Femoral fractures/1000 person-years (95% CI^†^)
Reference group									
Men	6,400	1,264,840.91	4.15 (4.03–4.28)	4,871	1,193,887.25	3.35 (3.23–3.46)	1,529	70,953.65	21.59 (20.59–22.74)
Women	26,828	2,202,984.72	9.92 (9.77–10.06)	19,174	1,967,735.28	7.53 (7.39–7.66)	7,654	235,249.44	32.88 (32.14–33.64)
PD treatment									
Model I (possible PD)									
Men	378	26,501.79	13.74 (12.32–15.33)	155	16,949.31	8.07 (6.73–9.68)	223	9,552.48	23.64 (20.67–27.04)
Women	1,255	55,084.97	22.47 (21.17–23.85)	593	33,002.00	15.78 (14.34–17.38)	662	22,082.97	30.40 (28.16–32.83)
Model II (probable PD)									
Men	234	15,701.44	14.47 (12.60–16.62)	92	9,672.50	8.80 (7.00–11.07)	142	6,028.95	23.72 (20.01–28.11)
Women	685	27,588.01	24.72 (22.82–26.78)	327	15,328.23	19.10 (16.81–21.71)	358	12,259.78	29.59 (26.65–32.84)

PD: Parkinson’s diseas.

## Discussion

In this large-scale analysis of population-based data of a health insurance company we compared femoral fracture rates in older individuals with ‘possible’ or ‘probable’ PD to a reference group without PD. The rates of femoral fractures found in our analysis are comparable to rates of femoral fractures previously reported [26]–[28].Our data demonstrate that individuals aged 65 years or older with medically treated idiopathic PD have a more than doubled (men) or almost doubled (women) risk to sustain a femoral fracture compared to the reference group. To test the robustness of our results and the susceptibility to the inclusion and exclusion criteria, two models with refined definitions were used. ‘Possible’ PD (model I) showed lower hazard ratios compared to ‘probable’ PD (model II). Both models showed a similar picture with a higher hazard ratio in those PD patients without severe functional impairment compared to PD patients with severe functional impairment and higher hazard ratio in men compared to women.

In PD patients, one out of four falls results in an injury [6]. Falls and fractures represent the leading diagnosis for admission to hospital in PD patients [8]. Our finding of an increased risk for femoral fractures in PD is in line with previous studies that demonstrated an association of PD with an increased risk of falling and a decreased bone mineral density [10]. A meta-analysis pooling data from six prospective studies found 46% of PD patients to fall over a three month period [6]. In addition to known risk factors such as age, female gender and previous falls, various disease-specific risk factors are under discussion such as pathologic gait characteristics [5], [29], [30], balance problems [5], [29], impaired cognition [29], [31], and the use of dopaminergic drugs [5], [9].

However, the excess risk we found is lower than the values reported previously ranging from a 2.2- to a 4.6-fold risk [12], [14]–[16], [32]. Our study is based on an unselected population-based sample of individuals including all stages of PD and including community-dwelling older individuals as well as nursing home residents (in both, PD patients and the reference group). This approach is in clear contrast to a number of studies recruiting from either specialist clinics or cohort studies that excluded the frailest of these patients. Such an example of a non-representative sample would be the Osteoporotic Fractures in Men study (MrOS) that explicitly excluded men unable to walk without personal assistance [11], [33]. A similar approach was chosen for the recruitment of another cohort [14]. Considering the fact that the risk of falling increases with the disease stages [4], [6], the advantage of a population-based approach is apparent.

This study introduces an additional approach which may be useful for a better interpretation of the observed risk. We were able to stratify the cohorts according to the need for long-term nursing care as a functional parameter. About every other PD patient in our dataset required long-term nursing care. Stratifying our analyses by this indicator for severe functional impairment, we retrieved a more complex picture. PD patients without severe functional impairment had a more than two fold risk to sustain a femoral fracture, compared to the reference group. In contrast, PD patients with severe functional impairment did not (women) or only slightly (men) differ from the reference group with severe functional impairment. Although we could confirm that PD is a risk factor for femoral fractures, the above data indicate that not the diagnosis per se, but functional impairment as a consequence of PD contributes relevantly to the risk of femoral fractures. Severe functional impairment expressed as care need has been shown to be a risk factor for factures [34], [35] The analysis of PD patients versus a reference group presented in this study implies that not the underlying disease is attributable for this risk but the need for help with ADLs.

Our approach has several limitations that need to be considered when interpreting the findings. The dataset used does not allow diagnosis of PD with very high security. However, by introducing a ‘possible’ and ‘probable’ PD model (which showed comparable results) and by choosing definitions that make the inclusion of severe atypical Parkinsonism (with an even higher risk of falls) in the PD cohort very probable, we argue that our approach might rather over- than under-estimate the fracture risk in the PD group. Moreover, data from the Rotterdam study indicates that our model approach indeed has a high accuracy to detect the persons of interest: They found 78% of users of Parkinson medication to have clinically confirmed idiopathic PD; intake of more than one substance class yield 100% specificity [36]. The most prevalent other condition treated with dopaminergic drugs is the restless legs syndrome (RLS) [37]. Further arguments for the usefulness of the PD models introduced here are provided by results of epidemiological studies investigating treatment behavior of the most relevant “confounding” disease, i.e. RLS. These studies found that, contrary to what is recommended in the guidelines, only a minority of patients are treated with dopaminergic drugs [38]–[40].

Another shortcoming of our data is the lack of information on dosage. Data from Denmark demonstrated a dose-dependent association between fracture risk and Parkinson’s medication [15]. Reimbursement data in the dataset used in our study did not allow reliable calculation of dosage in the absence of regimes.

We identified persons as ‘possible’ PD (model I) and ‘probable’ PD based on medication records in 2004. We could not trace back the duration of dispensing for a longer time period. Hence, this study does not allow any conclusions with respect to the duration of PD.

Our data were derived only from one health insurance company and may not be representative for the whole German population. However, the AOK is the by far largest statutory health care insurance and is open to all people. In Bavaria, the AOK covers almost 50% of the population aged 65 years and older, thus we argue that this is indeed a representative sample.

## Conclusions

Our findings confirm the increased risk of femoral fractures in PD patients. However, according to our large population-based dataset it is lower than previously reported. Interestingly, diagnosis-specific differences in fracture risk was observable only in PD patients *without* severe functional impairment, whereas PD patients and the reference group *with* severe functional impairment did not show relevant differences in fracture risk. This may have relevant implications for disease-specific falls prevention programs in PD patients. Such programs may be most effective in early rather than in advanced stages of the disease.
